# Evaluation of the effect of oral taurine supplementation on fasting levels of fibroblast growth factors, β-Klotho co-receptor, some biochemical indices and body composition in obese women on a weight-loss diet: a study protocol for a double-blind, randomized controlled trial

**DOI:** 10.1186/s13063-019-3421-5

**Published:** 2019-05-31

**Authors:** Fatemeh Haidari, Maryam Asadi, Javad mohammadi-asl, Kambiz Ahmadi-Angali

**Affiliations:** 1 Department of Nutrition, Nutrition and Metabolic Diseases Research Center, Ahvaz Jundishapur University of Medical Sciences, Ahvaz, Iran; 2 Department of Medical Genetics, Faculty of Medicine, Ahvaz Jundishapur University of Medical Sciences, Ahvaz, Iran; 3 Faculty of Public Health, Ahvaz Jundishapur University of Medical Sciences, Ahvaz, Iran

**Keywords:** Taurine supplementation, Weight-loss diet, Fibroblast growth factors, Obesity

## Abstract

**Background:**

Taurine (Tau) is involved in many biochemical functions such as regulation of glucose and lipid metabolism, enhancement of energy expenditure, anti-inflammatory effects and appetite control. The most important effect of Tau in obesity is its direct effect on adipose tissue. Some evidence has shown an impaired FGF (fibroblast growth factor) 19 and 21 biosyntheses in obesity. Besides the effects of eicosapentaenoic acid on serum FGF concentrations, the effect of other nutrients on FGFs is not clear. Since obesity as an important health problem is rising around the world and on the other side, Tau biosynthesis is reduced by adipose-tissue-derived factors in obesity, the effects of Tau and a weight-loss diet on obesity need to be investigated further.

**Methods:**

We will conduct an 8-week. double-blind, parallel-group, randomized controlled clinical trial to investigate the effect of Tau supplementation on fasting serum levels of FGFs, β-Klotho co-receptor, some biochemical indices and body composition in 50 obese women aged between 18 and 49 years on a weight-loss diet.

**Discussion:**

We will determine the other advantages of a weight-loss diet on new metabolic risk factors. Since Tau may regulate adipose-tissue-derived factors and a weight-loss diet can promote the useful effects of Tau supplementation; for the first time, the effects of a weight-loss diet along with Tau supplementation on these variables will be assessed.

**Trial registration:**

Iran Clinical Trials Registry, ID: IRCT20131125015542N2. Registered on 24 November 2018.

**Electronic supplementary material:**

The online version of this article (10.1186/s13063-019-3421-5) contains supplementary material, which is available to authorized users.

## Introduction

Obesity rates are rising around the world. Since excess body weight is associated with a higher incidence of cardiovascular disease, hyperlipidemia, hypertension, type 2 diabetes mellitus and some cancers, obesity is the fifth cause of mortality all over the world [1, 2]. According to the new studies, some novel obesity-related hormones may have an important role in obesity treatment as metabolic regulators [3].

Fibroblast growth factors (FGFs) 19 and 21 as members of FGFs are different from canonical FGFs. FGF19 and FGF21 could circulate in vessels as hormones. They increase total energy expenditure. Furthermore, they may decrease blood glucose, insulin, triglycerides, fat mass and body weight [4]. FGF19 and FGF21 link to a unique dual receptor complex consisting of β-klotho and activate tyrosine kinase FGF receptors (FGFR1–4) via a low-affinity interaction with heparan sulfate glycosaminoglycans (HSGAGs). β-klotho links to those and facilitates FGFR activation. β-klotho mainly expresses in metabolic organs including liver, adipose tissue and pancreas [5, 6].

Some evidence has shown that FGF19 (released by the intestine) and FGF21 (released by the liver and adipose tissue) play an important role in glucose and lipid metabolism. Some evidence has also shown an impaired FGF19 and 21 biosyntheses in obesity [7]. According to the result of a cross-sectional study, the serum level of FGF21 and FGF19 was high and low in the obese subjects, respectively. Furthermore, β-klotho gene expression was decreased. Adipose-derived pro-inflammatory factors could decrease gene expression of β-klotho in obesity. Since the function of FGF19 and FGF21 is dependent on β-klotho as a co-receptor; decreased expression of β-klotho causes metabolic disorders [8]. Thus, β-klotho co-receptor has been considered as a new marker in metabolic diseases [9, 10]. Since β-klotho expression is affected by adipose-derived pro-inflammatory factors, weight loss could be effective in reduction of resistance to FGF21 and β-Klotho co-receptor up-regulation.

Adiponectin has an important role in glycemic and lipid homeostasis. Recent evidence has shown that FGF21 increases adiponectin expression [7]. Generally, the serum level of FGF21 is directly associated with insulin resistance and increases liver stress markers in obese individuals. In addition, the serum level of FGF19 is reversely associated with insulin sensitivity and improvement in lipid metabolism. It seems that these two FGFs overlap in the body metabolism function [8]. Although one study showed the increasing effect of eicosapentaenoic acid (EPA) on serum FGF21 [11], the effect of other nutrients on FGFs is not clear.

Taurine (2-aminoethanesulfonic acid, Tau) is a sulfur-containing amino acid that is synthesized endogenously from cysteine (Cys) or methionine (Met). Additionally, this amino acid could be provided by diet, especially seafood. Diet-derived Tau is carried in the blood circulation in a low amount to other tissues [12]. There is some evidence that shows that the serum level of Tau is reduced in obesity [13, 14]. Tau is involved in many biochemical functions such as regulation of glucose and lipid metabolism, enhancement of energy expenditure, anti-inflammatory effects and appetite control. The most important effect of Tau in obesity is its direct effect on adipose tissue [12]. Tau increases genes expression that is related to energy expenditure including peroxisome proliferator-activated receptor (PPAR) α, PPAR γ and PPAR γ co-activator protein (PGC)-1α. Thus, this amino acid increases energy expenditure in the white adipose tissue. In the other side, Tau induces PGC-1α gene expression in the brown adipose tissue [15]. Also, in adipose tissue, it decreases the number of M1 macrophages (secreting pro-inflammatory cytokines and reactive oxygen species (ROS)) and increases the number of M2 macrophages (involved in the clearance of free fatty acid (FFA) and inhibition of lipotoxicity). Hence, Tau could reduce inflammatory markers such as high-sensitivity C-reactive protein (hs-CRP) [12, 15]. Also, this amino acid is anti-inflammatory and anti-oxidant through its sulfonic acid group and non-participation in protein structure (in free-form) [16]. Although the result of studies is controversial, there is some evidence that shows the beneficial effect of Tau on energy expenditure, weight and body composition in obesity and diabetes in both animals and humans [15]. Tau has a putative role in increasing energy expenditure, fatty acid β-oxidation and adipose tissue hypertrophy reduction [15]. Since serum FGF concentration is associated with visceral obesity [7] and the effect of Tau supplementation on serum FGF concentration is not clear, we decided to conduct a randomized controlled clinical trial (RCT) investigating the effect of Tau supplementation on fasting serum levels of fibroblast growth factors (FGF19, FGF21), β-Klotho co-receptor, some of the metabolic risk factors and body composition in obese women on a weight-loss diet.

### Hypothesis and aims

We hypothesize that Tau supplementation along with a standard weight-loss diet improves serum levels of biochemical parameters and body composition. The primary aims of the present RCT are the assessment of Tau supplementation on fasting serum levels of biochemical parameters and body composition. Furthermore, the secondary aims will evaluate the associations between changes in concentrations of fibroblast growth factors (FGF19, FGF21) and β-Klotho co-receptor with other variables.

## Methods

### Design and setting

**Fig. 1:**
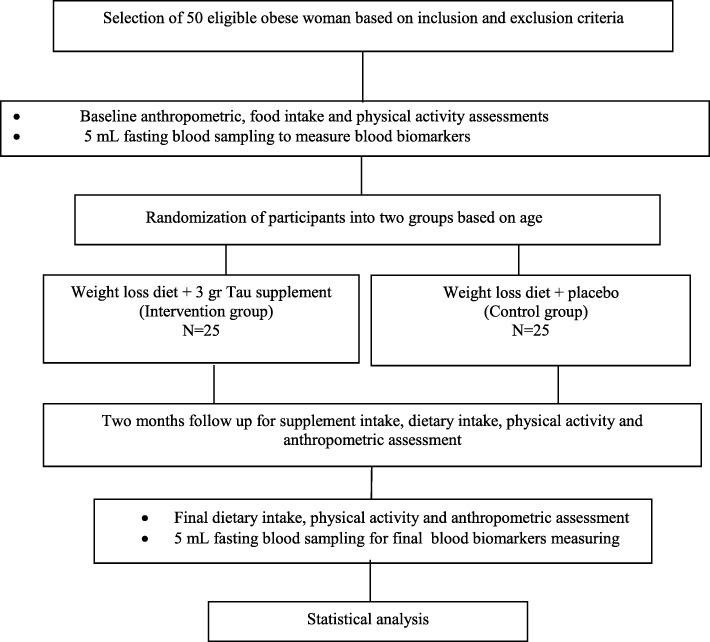
Flow diagram of the study

We will be performed a double-blind, parallel-group, clinical RCT. The proposed RCT will be conducted at the Private Nutrition Therapy Clinics in Ahvaz for 8 weeks to evaluate the effect of daily 3-g Tau supplementation in obese individuals Fig. 1. (The Standard Protocol Items: Recommendations for Interventional Trials (SPIRIT) 2013 Statement will be followed in this trial (Additional file 1)). Figure 1 shows the Flow diagram of the study.

### Participants

Participants will be 50 non-menopause obese women. Inclusion criteria will be included women aged 18 to 49 years old; body mass index (BMI) range between 30 and 40 kg/m^2^; exclusion criteria will be included: menopause, pregnancy and lactation; having a history of food allergy, cancer, acute or chronic renal failure, acute or chronic hepatic failure, thyroid disorders, gastrointestinal diseases, taking multivitamin/mineral supplements, taking herbal supplements or weight-loss drugs, surgery for weight loss and any weight loss over the past 6 months.

### Ethics and trial registration

The eligible participants will be notified about the study protocol. The protocol is approved by the Ethics Committee of Ahvaz Jundishapur University of Medical Sciences that is in accordance with the Declaration of Helsinki (approval number: IR.AJUMS.REC.1397.590). Each participant will sign an informed consent form. All collected data will be held confidential. This study is registered with the Iranian Registry of Clinical Trials (registration number: IRCT20131125015542N2).

### Sample size

The sample size was calculated based on the effect of Tau supplementation on changes in hs-CRP in obese people that was conducted by Rosa et al. [12]. It was computed by considering 95% confidence interval and 80% power (α = 0.05 and β = 0.2). In addition, the mean and standard deviation (SD) of hs-CRP levels in the mentioned study was as follows: μ_1_ = 14.30; μ_2_ = 10.50; SD_1_ = 2.90; SD_2_ = 2.40. We considered a 20% attrition rate. Finally, 25 subjects are considered for each group. All individuals will be included in the RCT if they meet the inclusion criteria and are willing to participate in the study to achieve the estimated sample size.

### Randomization and blinding

Eligible subjects will be divided and stratified based on age (within 10-year intervals) randomly into two groups including control (standard weight-loss group + taking placebo, *n* = 25) and intervention (standard weight-loss group + Tau supplementation, *n* = 25). Randomization will be performed using the computer-generated random numbers by a third party to reduce the probable bias. The third party will generate a random block design in blocks of 10.The naming of Tau or placebo bottles will be done based on random numbers and odd or even numbers will be allocated randomly to groups A or B. To preserve the blindness in case of any side effects, the third party will use unique codes instead of A or B.To achieve blinding, the bottles will be sealed, and we will be assured from the similarity of appearance and their weight. The researcher and participants will be blinded to the treatment allocation. Randomization codes of the RCT will be unlocked only after all individuals complete the study protocol.

### Intervention

**Fig. 2:**
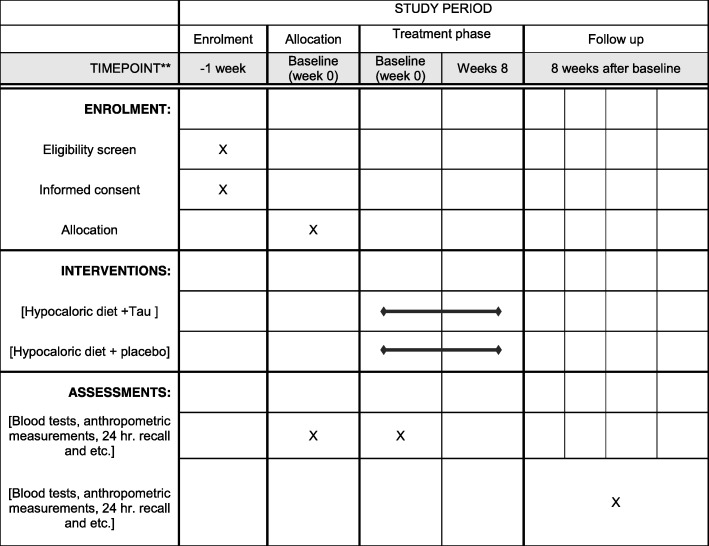
Schedule for enrollment, intervention and assessment based on Standard Protocol Items; Recommendations for Interventional Trials (SPIRIT) Figure

All subjects will follow a hypocaloric diet, whose energy needs will calculated by the Mifflin St. Jeor equation. Then, 30% of estimated energy requirements will deduct. The intervention group will receive 1-g Tau capsule three times a day after breakfast, lunch and dinner. The Tau supplementation dose will be determined according to the previous study [12].Tau supplement will be provided by Nutricost Company (Midvale, UT, USA). In addition, Placebo capsules will be provided by the Pharmacy Faculty of Ahvaz Jundishapur University of Medical Sciences in the same form and size as the Tau capsules. All capsules will be given to participants in the similar packing every 15 days. The macronutrients of a hypocaloric diet will be 50% carbohydrate, 30% fat and 20% protein. Considering the general principles of dieting, a trained dietitian will give a dietary exchange list and an individualized diet according to the subjects’ dietary habits. The same dietitian will follow the subjects to check compliance through phone calls or SMS every 3 days. Figure 2 shows the schedule for enrollment, intervention and assessment based on the Standard Protocol Items; Recommendations for Interventional Trials (SPIRIT) Figure.

### Measurements

An individual information questionnaire including demographic situations, history of diseases, supplementations and medications will be filled at the baseline. Dietary intake will be evaluated by 3-day 24-h recall questionnaires (two weekdays and one weekend day) at the beginning, middle and the end of the study. Total calorie and macroniutrients intake will be calculated using Nut IV (the Hearst Corporation, San Bruno, CA, USA ). The participants will be asked not to change their physical activity level (PAL). Physical activity levels will be assessed by the International Physical Activity Questionnaire (IPAQ) at the beginning and end of the study. The physical and anthropometric measurements will be given with minimal clothing and without shoes. Body weight will be measured using a 100-g accuracy scale (Seca). Height will be measured using a 0.5-cm accuracy Seca stadiometer. BMI will be computed by dividing body weight (kg) to the square of height (m). Waist circumference will be measured using a tape meter at the midpoint between the lowest rib and iliac crest and at the end of a normal expiration to the nearest 0.5 cm. A TANITA BC-418 body analyzer will be applied to estimate total body fat, fat percent, fat-free mass and fat-free mass percent. Five milliliters of venous blood sample (in a regular tube) will be taken after a 10–12-h overnight fast at the baseline and end of the study. Blood samples will be centrifuged at 1500 g for 15–20 min to separate the serum. Serum will be stored at − 80 °C and will be used for biochemical analysis, such as FGF19 (μg/mL), FGF21 (μg/mL), β-klotho co-receptor (μg/mL), leptin (μg/mL), adiponectin (μg/mL), hs-CRP (μg/mL), insulin (μU/mL), fasting blood sugar (FBS) (mg/dL), total cholesterol (TC), high-density lipoprotein (HDL-C), triglyceride (TG), alanine transferase (ALT), aspartate transaminase (AST) and gamma-glutamyl transferase (GGT), in the serum. Enzyme-linked immunosorbent assay (ELISA) kits will be applied to measure serum FGFs, β-klotho co-receptor, leptin, adiponectin, hs-CRP and insulin, lipid profile and serum glucose concentration. Also, serum hepatic enzymes will be measured using the enzymatic method by Pars Azmoon kits (Tehran, Iran). Low-density lipoprotein (LDL-C) concentrations will be computed by the Friedewald equation. Homeostasis model assessment – insulin resistance (HOMA-IR) will be calculated as follows:

$$ \mathrm{FBS}\ \left(\mathrm{mg}/\mathrm{dL}\right)\times \mathrm{fasting}\ \mathrm{serum}\ \mathrm{insulin}\ \left(\mu \mathrm{U}/\mathrm{mL}\right)/405, $$

where FBS is fasting blood sugar.

### Statistical analysis

We will use intention-to-treat (ITT) and per-protocol (PP) populations in the analysis. The ITT population consists of all individuals who will be randomized, whereas the PP population consists all participants who complete the 8-week intervention. The data will be revised randomly to check accuracy and completeness. All data will be reported as mean ± SD. The percentage changes for each variable will be computed by the following formula:

$$ \left[\left(E-B\right)/\mathrm{B}\times 100\right], $$

where *E* and *B* are the end value and the baseline value of variable, respectively. The data normality will be analyzed using the Kolmogorov-Smirnov test. To compare parametric continuous data between and within the groups, the independent sample *t* test and the paired sample *t* test will be used, respectively. In addition, to compare the differences in asymmetric variables between and within the groups, the Mann-Whitney test and Wilcoxon test will be applied, respectively. The analysis of covariance (ANCOVA) test will be used to control confounding variables. To evaluate the association between changes in fibroblast growth factors (FGF19, FGF21), β-Klotho co-receptor concentrations and other variables, linear regression models will be used. SPSS version 21 (IBM, Armonk, NY, USA) will be used for data analysis. A *p* value < 0.05 will be considered statistically significant.

### Safety, adverse effects and monitoring data

There are no known side effects for 3-g/day Tau supplementation [12]. However, this RCT will supervise by a Data Monitoring Committee (DMC). In addition, any possible side effects will be reported to the Ethics Committee of the Ahvaz University of Medical Sciences.

## Discussion

The discovery of the FGFs (FGF19, FGF21) and their influences on the body energy balance as hormones demonstrate significant progress in obesity and type 2 diabetes studies. It seems that FGF21, FGF19 and β-klotho concentrations are correlated with risk factors for metabolic diseases especially in subjects with abdominal obesity. Weight loss may diminish obesity risk via regulation of adipose-tissue-derived factors, finally modulating concentrations of FGFs (FGF19, FGF21) and β-klotho co-receptor. In addition, according to the studies, Tau may regulate adipose-tissue-derived factors. Therefore, a weight-loss diet can promote the useful effects of Tau supplementation. This study should determine another beneficial effect of Tau supplementation through regulation of FGFs and β-klotho co-receptor along with a standard weight-loss diet.

## Additional file

Additional file 1: Standard Protocol Items: Recommendations for Interventional Trials (SPIRIT) 2013 Checklist: recommended items to address in a clinical trial protocol and related documents. (DOC 122 kb)

## Data Availability

The results will not be available before publishing.

## Abbreviations

ALT: Alanine transferase
ANCOVA: Analysis of covariance
AST: Aspartate transaminase
BMI: Body mass index
Cys: Cysteine
EPA: Eicosapentaenoic acid
FBS: Fasting blood sugar
FFA: Free fatty acid
FGFR: FGF receptors
FGFs: Fibroblast growth factors
GGT: Gamma-glutamyl transferase
HDL-C: High-density lipoprotein
HOMA-IR: Homeostasis model assessment – insulin resistance
hs-CRP: High-sensitivity C-reactive protein
IPAQ: International Physical Activity Questionnaire
ITT: Intention to treat
LDL-C: Low-density lipoprotein
Met: Methionine
PAL: Physical activity level
PGC: PPAR γ co-activator protein
PPAR: Peroxisome proliferator-activated receptor
RCT: Randomized controlled clinical trial
ROS: Reactive oxygen species
Tau: Taurine
TC: Total cholesterol
TG: Triglyceride
